# Weighted Gene Correlation Network Analysis Reveals Key Regulatory Genes Influencing Selenium Enrichment and Yield with Exogenous Selenite in Tartary Buckwheat

**DOI:** 10.3390/plants14030423

**Published:** 2025-02-01

**Authors:** Xueling Ye, Linsen Mei, Zhen Gan, Zhiqiang Wang, Wenjun Sun, Yu Fan, Changying Liu, Qi Wu, Yan Wan, Xiaoyong Wu, Dabing Xiang

**Affiliations:** 1 Key Laboratory of Coarse Cereal Processing, Ministry of Agriculture and Rural Affairs, Sichuan Engineering & Technology Research Center of Coarse Cereal Industralization, School of Food and Biological Engineering, Chengdu University, Chengdu 610106, China; shirlingye@gmail.com (X.Y.);; 2 Animal Husbandry and Fishery Equipment Research Center, Sichuan Academy of Agricultural Machinery Sciences, Chengdu 610066, China; 3 Institute of Agronomy and Horticulture, Chengdu Agricultural College, Chengdu 611130, China

**Keywords:** *Fagopyrum tataricum* (L.) Gaertn., transcriptome, absorption and transport of selenite, differentially expressed genes, regulation model

## Abstract

Selenium (Se) is an essential trace element for human health, and dietary Se intake is an effective supplement. Rich in nutrients and functional components with potential for Se enrichment, Tartary buckwheat (*Fagopyrum tataricum* (L.) Gaertn.) is a Se-biofortified cereal. To determine the optimal Se treatment concentration and fully understand its effects on Tartary buckwheat, sodium selenite (Na_2_SeO_3_) in different concentrations was sprayed onto leaves of Tartary buckwheat at the initial flowering stage. Agronomic and yield-related traits and Se enrichment were analyzed between CK and treatments. The results showed that Na_2_SeO_3_ concentrations of 3.0 and 6.0 mg/L significantly increased the contents of Se and starch in the grains, the 1000-grain weight, the number of grains per plant, and the yield. The 6.0 mg/L treatment had the best effect. Transcriptome and weighted gene co-expression network analyses showed that selenite promoted chlorophyll synthesis and photoelectron transport by upregulating chlorophyll synthase (CHLG) and protein CURVATURE THYLAKOID 1B (CURT1B) levels, improving photosynthesis, increasing sucrose synthesis and transport in leaves and starch synthesis and accumulation in grains, and promoting grain-filling and yield. These changes were regulated by genes related to photosynthesis, sucrose, and starch metabolism-related genes, including *CAB3C*, *HPR3*, *SUS5*, *BAM9*, *SS3*, *SWEET1*, and *SWEET12*. Selenite absorption in Tartary buckwheat was regulated by aquaporin genes *NIP1-1* and *PIP1-5*. Selenite transport was regulated by the inorganic phosphate transporter gene *PHT1-1*, and organic Se transport was controlled by the proton-dependent oligopeptide transporters NPF3.1 and NPF4.6. Methionine gamma-lyase (MGL) was involved in selenocompound metabolism. This study identified the best spraying scheme for enhancing Se content in the grains. It also revealed the regulatory genes responding to selenite absorption, transport, and metabolism and the regulatory pathways promoting yield in Tartary buckwheat. These results provide technical guidance and theoretical support for producing high-yielding and Se-enriched Tartary buckwheat.

## 1. Introduction

Selenium (Se) is an essential micronutrient for human health. It has multiple biological effects, such as antioxidation, free radical scavenging, immune function enhancement, and cancer cell division and growth prevention [1]. Studies have demonstrated that Se can effectively treat Keshan disease, Kaschin–Beck disease, cardiovascular and cerebrovascular diseases, etc. [2]. In 1988, the Chinese Nutrition Society listed Se as one of the 15 nutrients beneficial to human health. According to the recommendations of the World Health Organization (WHO), the daily recommended intake of Se for adults is 50–200 ug. However, around 15% of the world’s population is Se deficient, and more than 100 million people in China have health problems caused by Se deficiency [3,4]. Se intake is related to the dietary structure [5]. Some issues related to nutritional Se deficiency are due to unbalanced and overprocessed diets and a lack of whole and coarse grains [6]. Plant-based foods are the main source of Se supplementation in humans and have many advantages such as being natural, safe, nutritionally complete, and having high ecological value [5]. Therefore, converting inorganic Se to plant-based Se through crop fortification effectively increases people’s dietary Se intake, providing health benefits and resource efficiency [5,7]. Tartary buckwheat (*Fagopyrum tataricum* (L.) Gaertn.), an annual plant in the Polygonaceae family, has beneficial properties as both food and medicine. It is rich in nutrients and functional components, especially flavonoids and amino acids, which can lower blood pressure, lipids, and sugar and enhance human immunity [8]. Different from many other cereals, Tartary buckwheat is gluten free. It is very important and beneficial for celiac disease patients who are intolerant to gluten. Importantly, Tartary buckwheat grains contain Se, which is lacking in other cereals. It has excellent Se-enrichment potential and can serve as an important crop source for Se supplementation of the human body.

Se not only has health benefits for humans but also improves plant growth and development. As an important element, Se participates in the active center of glutathione peroxidase (GPx), which can improve the antioxidant effects of plants and enhance stress resistance [9,10]. Many studies have reported Se enrichment in crops, such as rice, maize, and wheat. Ramos et al. (2023) found that foliar Se application significantly increased the chlorophyll content, grains per panicle, 100-grain weight, and yield in rice [11]. Se application in wheat increased photosynthesis, yield, and Se content of grains, and improved Cd stress tolerance [12,13]. In maize, Se application effectively increased contents of Se and chlorophyll, stem growth, plant height, 1000-grain weight, and yield under reasonable planting density [14]. In addition, foliar Se application effectively improved starch accumulation and the quality of millet grain by enhancing the activities of enzymes related to grain starch synthesis [15]. Se positively affected photosynthetic pigments, seed number, and the yield of cowpea and improved sucrose starch accumulation in mung bean [16,17]. In Tartary buckwheat, appropriate Se application effectively promoted Se content in grains and increased yield [18]. Se also increased the photochemical efficiency of photosystem II and electron transport system activity in Tartary buckwheat [19]. Ma et al. (2023) and Cao et al. (2021) reported that Se effectively promoted photosynthesis, yield, quality, and antioxidant activity in Tartary buckwheat [20,21].

Plants absorb Se through their roots and leaves. The roots mainly absorb inorganic Se and a few organic Se forms (SeCys, SeMet) in the soil, while the leaves mainly absorb inorganic, organic, and nano-elemental Se through stomata. Se has multiple valence states in the environment, among which Se^6+^ and Se^4+^ (selenate and selenite, respectively) are the two main forms, and they are easily absorbed by plants. The selenates enter plants through an energy-driven sulfate transport system [22,23]. Most selenates enter the chloroplast and are catalyzed to form selenite and then enter the same metabolic pathway as absorbed selenite. Part of them form organic Se such as seleno-amino acids, selenoproteins, and selenopolysaccharides, and part of them is reduced to inorganic Se such as selenite and selenide [24]. Relatively few studies have evaluated selenite absorption. Previous studies have shown that selenite is mainly absorbed by passive transport, and phosphate transporters, silicon transporters, and aquaporins may be involved in selenite absorption and transport [23]. Zhao et al. (2010) first confirmed that selenite absorption was mediated by the silicon transporter OsNIP2;1 in rice [25]. The phosphate transporter OSPT2 is involved in selenite absorption in rice [26]. In tobacco, phosphate transporter OSPT8 mediates selenite absorption [27]. In mung bean, low sodium selenite concentrations promote germination and growth and effectively increase the dry and fresh weight of sprouts. Se (IV) in the cotyledons enters the buds and roots regulated by *PHT1.1*, *NIP2*, and *SULTR3;3* [28]. However, no studies have reported the absorption-mediated proteins and regulatory genes of Se in Tartary buckwheat.

The production of selenium-enriched Tartary buckwheat is not only beneficial to human health but also increases its added value. Results from various species show that Se content is affected by the application concentration and method as well as genotypes. However, relevant research on Tartary buckwheat is limited. No molecular studies have reported on Se content in Tartary buckwheat or its effects on yield. Therefore, in this study, physiological traits, agronomy, yield-related traits, Se content in grains, and gene expression were investigated in Tartary buckwheat under Na_2_SeO_3_ treatments. The objectives were to (1) identify the optimal concentration for Se-enriched treatment, (2) investigate the comprehensive influences of selenite on Tartary buckwheat, and (3) analyze the key regulatory genes of Se application on yield increase and Se accumulation in Tartary buckwheat. Results from this study provide scientific technical guidance and a theoretical basis for researching and breeding high-yielding and Se-enriched Tartary buckwheat varieties.

## 2. Results

### 2.1. Photosynthetic Pigment Contents

**Figure 1:**
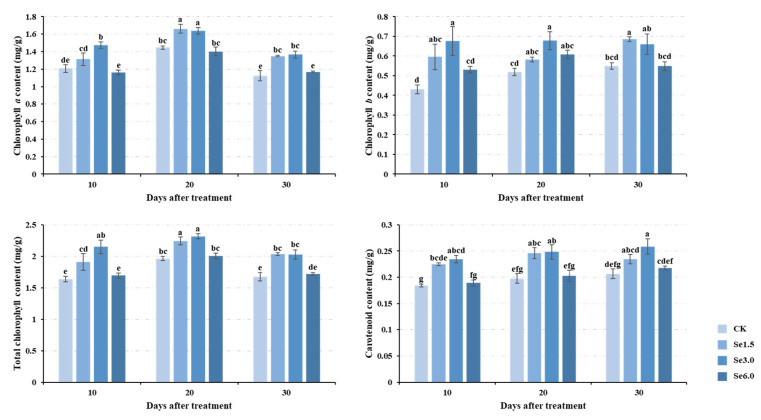
Changes in chlorophyll *a*, *b*, total chlorophyll, and carotenoid contents in Tartary buckwheat on days 10, 20, and 30 following selenite treatment. Different lowercase letters above the bars indicate significant differences between the means according to Duncan’s test (*p* ≤ 0.05). CK, Se1.5, Se3.0, and Se6.0 indicate the Na_2_SeO_3_ concentrations 0, 1.5, 3.0, and 6.0 mg/L.

Photosynthetic pigments are closely related to photosynthesis in plants and are indispensable in higher plants. The contents of four photosynthetic pigments under different treatments were measured 10, 20, and 30 days after spraying Na_2_SeO_3_. Na_2_SeO_3_ treatment increased the photosynthetic pigment content. With the same Na_2_SeO_3_ treatment concentration, the chlorophyll (Chl) *a* and total Chl contents first increased and then decreased over time, peaking on the 20th day. The carotenoid (Car) and Chl *b* contents showed an upward trend over time but were insignificant (Figure 1). The contents of the four photosynthetic pigments first increased and then decreased with a treatment concentration of Na_2_SeO_3_, regardless of the number of days after treatment (Figure 1). The Chl *a* and Chl *b* contents reached the highest levels with Se1.5 (1.5 mg/L Na_2_SeO_3_) treatment on the 20th and 30th days, respectively. The total Chl and Car contents were highest with the Se3.0 (3.0 mg/L Na_2_SeO_3_) treatment on the 20th and 30th days, respectively (Figure 1). The total Chl and Car contents increased by 17.83% and 25.08% compared to CK.

### 2.2. Sucrose and Starch Contents and GPx Activity in Leaves and Grains

**Figure 2:**
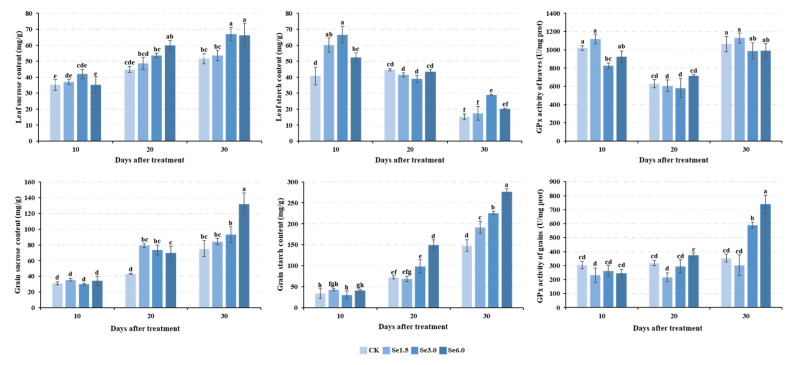
Changes in the sucrose and starch contents and GPx activity of Tartary buckwheat on days 10, 20, and 30 following selenite treatment. The three bar graphs above show the sucrose and starch contents and GPx activity in leaves, and the graphs below show the sucrose and starch contents and GPx activity in grains. Different lowercase letters above the bars indicate significant differences between the means according to Duncan’s test (*p* ≤ 0.05). GPx, glutathione peroxidase. CK, Se1.5, Se3.0, and Se6.0 indicate the Na_2_SeO_3_ concentrations 0, 1.5, 3.0, and 6.0 mg/L.

To understand the effects of Na_2_SeO_3_ treatment on photosynthetic product accumulation and GPx activity of Tartary buckwheat, sucrose and starch contents and GPx activity were tested in leaves and grains at 10, 20, and 30 days after treatment with different Na_2_SeO_3_ concentrations. Under the same Na_2_SeO_3_ treatment concentration, the sucrose content of the leaf and grain and starch content of grain generally increased over time. At 20 and 30 days after treatment, their contents increased as the treatment concentration increased. The leaf sucrose content of the Se3.0 treatment and the grain sucrose and starch contents of the Se6.0 (6.0 mg/L Na_2_SeO_3_) treatment were highest on the 30th day and were 29.79, 75.35, and 86.71% higher than those of CK (0 mg/L Na_2_SeO_3_), respectively (Figure 2). The same Na_2_SeO_3_ treatment decreased the starch content in the leaves over time. On the 10th day after treatment, the starch content first increased and then decreased with the increase in the Na_2_SeO_3_ concentration, and it was highest under the Se3.0 treatment (Figure 2).

Under the same Na_2_SeO_3_ treatment, the GPx activity of the leaves first decreased and then increased over time. On the 10th day, the GPx activity was significantly lower under the Se3.0 treatment than under the other treatments. No significant differences were observed between treatments on the 20th or 30th days (Figure 2). In grains, there were no significant differences between treatments on the 10th and 20th days after Na_2_SeO_3_ treatment. On the 30th day, the GPx activity was significantly higher under the Se3.0 and Se6.0 treatments than under the other treatments, and Se6.0 showed the highest values, increasing by 100.09% compared to that of CK (Figure 2).

### 2.3. Agronomic, Yield-Related, and Quality Traits and Se Concentrations in Mature Grains

**Figure 3:**
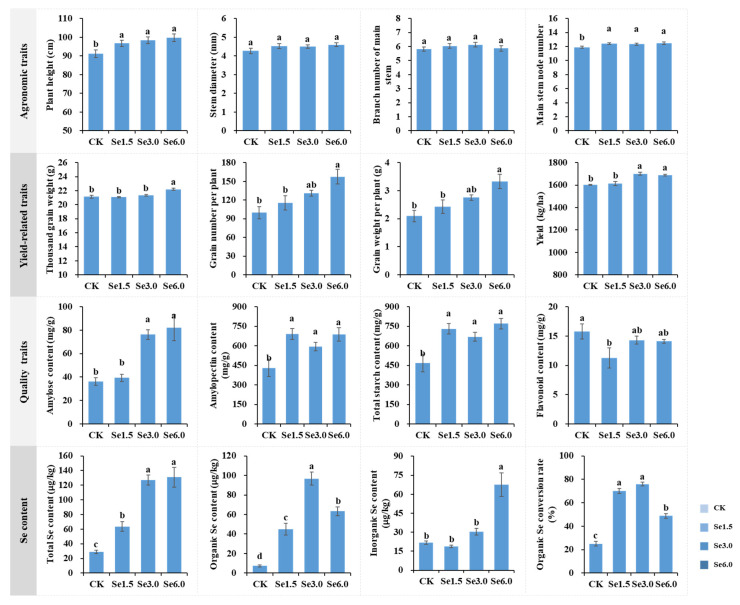
Agronomic, yield-related, quality traits, and Se enrichment of Tartary buckwheat between control and selenite treatments. Different lowercase letters above the bars indicate significant differences between the means according to Duncan’s test (*p* ≤ 0.05). CK, Se1.5, Se3.0, and Se6.0 indicate the Na_2_SeO_3_ concentrations 0, 1.5, 3.0, and 6.0 mg/L.

Plant height and the main stem node number increased significantly after Na_2_SeO_3_ treatment compared to those of CK. There were no significant differences in the stem diameter and branch number on the main stem among the treatments (Figure 3). Na_2_SeO_3_ treatment enhanced the yield-related traits. The 1000-grain weight, grain number per plant, and grain weight per plant were significantly higher under the Se6.0 treatment than under other treatments. The yield under the Se3.0 and Se6.0 treatments was significantly higher than that under the other treatments and had values 6.16 and 5.34% higher than that under CK, respectively (Figure 3).

Amylopectin and total starch contents significantly increased under the Na_2_SeO_3_ treatment compared with CK (Figure 3). The amylose content under the Se3.0 and Se6.0 treatments was higher than that under the other treatments. The flavonoid content decreased under the Se1.5 treatment, and there were no changes under the other treatments (Figure 3).

Exogenous Na_2_SeO_3_ treatment significantly increased Se contents in Tartary buckwheat grains (Figure 3). The overall trend in the Se content showed an increase with the increase in the treatment concentration. The total Se content of the Se6.0 treatment reached a maximum of 130.8 μg/Kg. The organic Se content of the Se3.0 and Se6.0 treatments significantly increased, and that of the Se3.0 treatment was 12.5 times higher than that of CK. The Se1.5 and Se3.0 treatments had the highest organic Se conversion rates of 70.1 and 74.5%, respectively. Considering both yield and Se contents, we conclude that the treatment with 6.0 mg/L Na_2_SeO_3_ is optimal.

### 2.4. Quality Control of the Transcriptome Sequence

The leaves and grains of the Se6.0 treatment and CK at 10, 20, and 30 days after spraying Na_2_SeO_3_ were selected for transcriptome sequencing with three biological replicates. After removing low-quality reads, a total of 236.70 Gb clean reads were obtained, with clean reads of each sample over 5.74 Gb. The percentage of Q30 bases was 93.17% or above, and the GC percentage was between 44.41 and 47.22% (Table S1), indicating that the quality of the transcriptome data was sufficient for further analysis. The Pearson correlation coefficients of the three repeated samples were all greater than 0.8, and the correlation coefficients of the repeated samples were higher than those of the non-repeated samples (Figure S1). This suggests good repeatability in the sequencing samples and that the transcriptome sequencing results are reliable.

### 2.5. Differentially Expressed Genes (DEGs) and Kyoto Encyclopedia of Genes and Genomes (KEGG) Pathway Analyses

**Figure 4:**
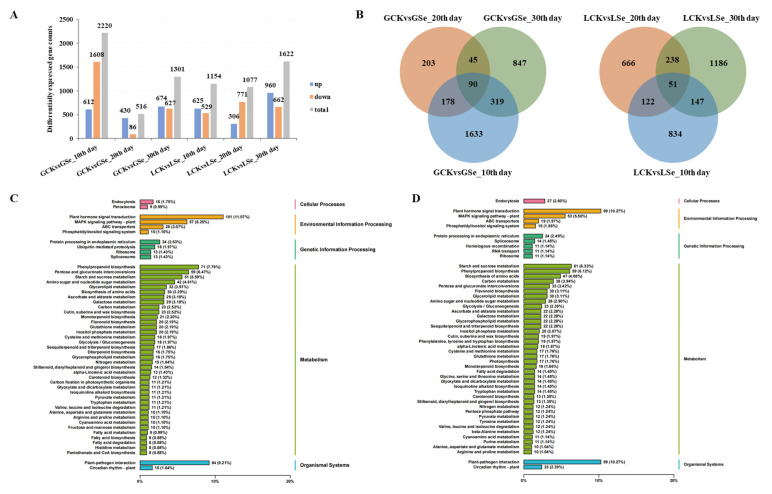
Histogram, Venn diagram, and KEGG enrichment analysis of the differentially expressed genes (DEGs) between the control and selenite treatments. (**A**) Histogram of the DEG counts. (**B**) Venn diagram of the DEGs. (**C**) KEGG enrichment analysis of DEGs in grains. (**D**) KEGG enrichment analysis of DEGs in the leaves. KEGG, Kyoto encyclopedia of genes and genomes; GCK, control of grains; GSe, grains with Se6.0 treatment; LCK, control of leaves; LSe, leaves with Se6.0 treatment; 10th day, 20th day, and 30th day indicate the days after selenite treatments.

Based on the Tartary buckwheat reference genome sequence, 40,184 genes were obtained from the transcriptomic sequences. They included 8525 novel genes, of which 4761 were functionally annotated. A total of 5639 DEGs, including 1516 novel ones, were identified in grains and leaves at different times between CK and Se6.0 and were identified according to the screen criteria of FC (fold change) ≥ 2 and FDR (false discovery rate) < 0.01. There were 2220, 516, 1301, 1154, 1077, and 1622 DEGs in the six pairwise comparisons of CK vs. Se6.0 in grains and leaves on the 10th, 20th, and 30th days, respectively (Figure 4A). Of these, 90 and 51 DEGs in the grains and leaves were all expressed on the 10th, 20th, and 30th days (Figure 4B).

Based on the KEGG pathway enrichment analysis, 3315 and 3244 DEGs were detected in grains and leaves, respectively. These were divided into five categories: cellular processes, environmental information processing, genetic information process, metabolism, and organismal systems (Figure 4C,D). In the environmental information processing category, the DEGs in grains and leaves were mainly enriched in the plant hormone signal transduction, the MAPK signaling pathway plant, and the ABC transporter pathways. In the genetic information process category, the DEGs in grains were mainly associated with protein processing in the endoplasmic reticulum and in ubiquitin-mediated proteolysis, and the DEGs in leaves were primarily related to protein processing in the endoplasmic reticulum and spliceosome. In the metabolism category, the DEGs in grains were mainly involved in phenylpropanoid biosynthesis, pentose and glucuronate interconversions, starch and sucrose metabolism, amino sugar and nucleotide sugar metabolism, and glycerolipid metabolism. The DEGs in the leaves were primarily enriched in starch and sucrose metabolism, phenylpropanoid biosynthesis, the biosynthesis of amino acids, carbon metabolism, and protein processing in the endoplasmic reticulum.

### 2.6. Weighted Gene Co-Expression Network Analysis (WGCNA) of Filtered Genes

**Figure 5:**
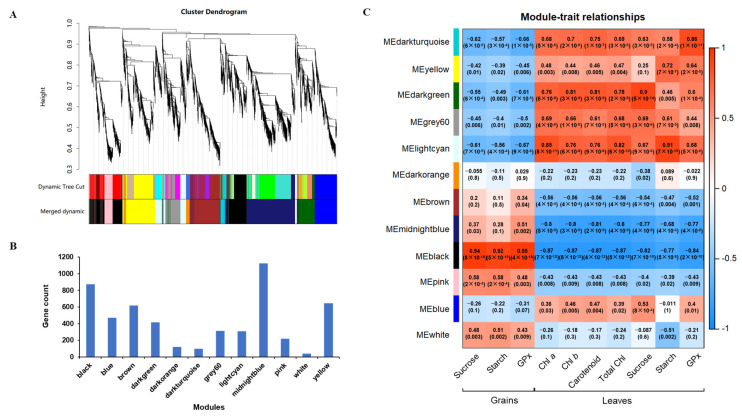
Weighted gene co-expression network analysis of physiological index in leaves and grains during the filling stage. (**A**) Gene cluster dendrograms and module detection. (**B**) Gene number for each module. (**C**) Heat map of module–trait correlation. The first three columns represent the traits in grains, while the subsequent seven columns correspond to the leaves. The different colors indicate different modules. The values in the brackets indicate the correlation coefficient (*p*-value) with a legend on the right. GPx, glutathione peroxidase; Chl, chlorophyll.

To identify genes affected by exogenous Se treatment in Tartary buckwheat, WGCNA was performed for genes with an FPKM (fragments per kilobase of transcript length per million mapped reads) value ≥ 0.01 and a variation in FPKM ≥ 0.5. The fitting index *R^2^* = 0.85 and the optimal soft threshold *β* = 6 were selected to guarantee a scale-free network (Figure S2). The clustering results of the co-expressed genes were dynamically cut, and 12 modules were obtained after combining similar clusters. The black module was positively associated with the sucrose and starch contents and GPx activity in grains, but negatively related to the photosynthetic pigment content in leaves. The dark green, light cyan, and dark turquoise modules were positively associated with the sucrose and starch contents and GPx activity in the leaves, respectively (Figure 5). Combined with the thresholds of module membership |MM| > 0.8 and gene significance |GS| > 0.8, the DEGs included in the hub genes with the top 100 levels of connectivity in each module were selected. There were 93, 29, 48, and 22 target DEGs in the black, dark green, light cyan, and dark turquoise modules, respectively. Combined with functional annotation, 20 candidate genes were identified (Table 1).

### 2.7. Real-Time Quantitative Polymerase Chain Reaction (qRT-PCR) Verification of Candidate Genes

**Figure 6:**
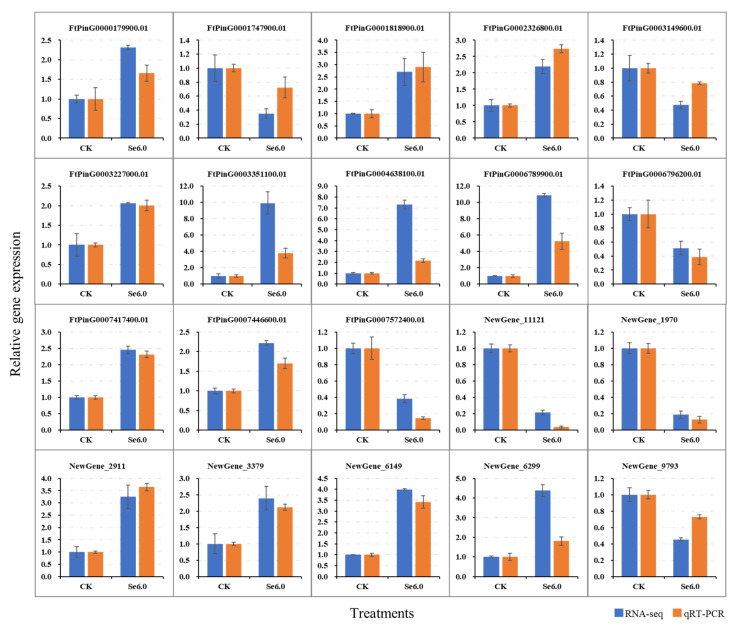
Comparison of relative gene expression of candidate genes based on RNA-seq and real-time quantitative PCR (qRT-PCR). CK and Se6.0 indicate the Na_2_SeO_3_ concentrations 0 and 6.0 mg/L.

Twenty candidate genes were selected for qRT-PCR validation to verify their expression levels. The primers for qRT-PCR analysis were designed according to their coding sequences (CDSs) (Table S2). The verification results are shown in Figure 6. The qRT-PCR expression trends in the 20 genes were consistent with the results of RNA sequencing (RNA-Seq). These results indicate that the candidate regulatory genes identified in this study are reliable and stable.

## 3. Discussion

### 3.1. Exogenous Se Effectively Promotes the Se Contents in Grains of Tartary Buckwheat Grains

The absorption and conversion capacity of Se vary among species and varieties [18,22]. Tartary buckwheat grains contain Se, which is lacking in other cereals. They have a natural Se-enriching capacity and great potential for Se enrichment. Research has shown that applying selenite to Tartary buckwheat leaves is a very effective method of Se biofortification [18,20,21]. This study showed that spraying Na_2_SeO_3_ on the leaves of Tartary buckwheat at the beginning of flowering effectively increased Se contents. As the treatment concentration increased, the total Se and inorganic Se contents of the grains increased (Figure 3). However, the organic Se content and organic Se conversion rate first increased and then decreased with the increase in treatment concentration, indicating an upper limit for Se absorption and the conversion efficiency of organic Se in Tartary buckwheat. Because the conversion rate of organic Se in plants is limited, it does not increase infinitely with the increase in the applied Se concentrations. If excessive inorganic Se is applied during the Se nutrition fortification process, there will be problems with inorganic Se residues in crops, risking poisoning and causing plant stress [24]. Therefore, the Se concentration used is critical for Se-enriched biofortification. In this study, through a comprehensive evaluation of the Se content and composition, the optimal Na_2_SeO_3_ treatment concentration on the leaves of Tartary buckwheat was 3.0–6.0 mg/L. These results lay a theoretical foundation for the production of Se-enriched Tartary buckwheat.

### 3.2. Candidate Genes Involved in the Regulation of Se Absorption, Translocation, and Metabolism

Selenite is mainly absorbed through passive transport. Phosphate transporters, silicon transporters, and aquaporins may be involved in selenite absorption and transport [23,24]. The selenite absorption and transport mechanisms are not clear. There is no report on Se absorption, transport, or metabolism-related regulatory genes in Tartary buckwheat. Here, we identified nine candidate genes that may be involved in Se absorption, transport, and metabolism in Tartary buckwheat (Table 1). Of them, three were novel.

FtPinG0003351100.01 and NewGene_2911 are the nodulin 26-like intrinsic protein 1-1 (NIP1-1) and plasma membrane intrinsic protein 1-5 (PIP1-5), respectively. They are aquaporins, involved in the transmembrane transport of water and small neutral solutes [29,30]. The aquaporins NIPs and PIPs have been shown to be involved in Se uptake [23,31]. In the present study, Na_2_SeO_3_ treatment was shown to significantly increase the expression levels of *NIP1-1* and *PIP1-5* (Table 1), which might have been attributed to selenite absorption. Zhao et al. (2010) found that the aquaporin OsNIP2;1 was related to selenite uptake in rice [25]. In maize, selenite uptake through aquaporins was affected by pH [32]. These studies supported our conclusions. FtPinG0007417400.01 is an inorganic phosphate transporter 1-1 (PHT1-1) which regulates inorganic ion and carbohydrate transport and metabolism [33,34]. Many studies have shown that the phosphate transporter is involved in selenite transport [35,36,37]. Zhang et al. (2014) demonstrated that selenite and Pi share similar uptake mechanisms and that the phosphate transporter OsPT2 is involved in selenite uptake [26]. The phosphate transporter gene *OsPT8* in tobacco has also been shown to be involved in selenite uptake based on overexpression analysis [27]. In the present study, *PHT1-1* was highly expressed under Na_2_SeO_3_ treatment. Therefore, PHT1-1 was likely associated with selenite transport in Tartary buckwheat. FtPinG0007572400.01 and NewGene_9793 are sulfate transporter 3.1 (SULTR3;1) and probable sulfate transporter 3.4 (SULTR3;4). These genes are known to be involved in sulfate and selenate transport [23,24]. Although SULTRs have been shown to regulate selenate transport, several studies have shown that they may also be related to selenite transport. Zhang et al. (2022) reported that several SULTRs were highly upregulated with selenite treatment in tea plants [38]. In mung bean cotyledons, Se (IV) passed through SULTR3;3 transport to shoots and roots [28]. The expression of *JrSULTR1.2b*, *JrSULTR1.2a*, and *JrSULTR3.2b* was downregulated under treatment with 0.8 mmol·L^−1^ sodium selenite in walnuts [39]. Similarly, in this study, *SULTR3;1* and *SULTR3;4* were downregulated expression under Na_2_SeO_3_ treatment in Tartary buckwheat. Thus, we speculated that SULTR3;1 and SULTR3;4 not only participate in selenate transport but also may affect selenite transport in Tartary buckwheat. FtPinG0000179900.01 and NewGene_3379 are the NRT1/PTR family proteins NPF4.6 and NPF3.1, respectively. They are known to participate in nitrate assimilation, amino acid transport, and metabolism [40,41]. The present study showed that Na_2_SeO_3_ treatment upregulated the expression levels of *NPF4.6* and *NPF3.1*, which may be related to organic Se (including Se-amino acid) transport in Tartary buckwheat. This conclusion coincides with previously reported research. Zhang et al. (2019) demonstrated that the nitrate transporter 1.1B (NRT1.1B) belonging to the peptide transporter (PTR) family has SeMet transport activity and mediates the root-to-shoot translocation of SeMet in rice [42]. In tea plants, the oligopeptide transporters have been shown to be involved in HSe transportation in response to Na_2_SeO_3_ [43]. In addition, PTR4, belonging to the NRT1/PTR family, was implicated in transporting Se amino acids from roots to shoots in dandelions [41]. FtPinG0001747900.01 is methionine gamma-lyase (MGL), which has been associated with selenocompound metabolism and cysteine and methionine metabolism [44,45]. In *Cardamine violifolia*, MGL was shown to be related to Se metabolism [46]. In alfalfa, MGL participated in Se metabolism under Na_2_SeO_3_ treatment [47]. In the study reported here, Na_2_SeO_3_ treatment resulted in the downregulation of MGL, indicating that MGL is associated with Se metabolism in Tartary buckwheat.

As an important element, Se participated in the formation of the active center of GPx, improving the antioxidant effect in plants and enhancing stress resistance in plants [9,10]. In the late filling period, the GPx activity of grains treated with Se3.0 and Se6.0 was significantly higher than that of CK, and many indicators were significantly improved, indicating that Se improves antioxidant properties by increasing GPx activity, which is beneficial for the growth and development of Tartary buckwheat. In *Pteris vittata*, selenate effectively increased plant growth via the GPX-GR-mediated enhancement of the GSH-GSSG cycle [48]. Similarly, the foliar Se application promoted plant growth and increased GPx activity [49]. The highly expressed gene FtPinG0007446600.01, a 1-Cys peroxiredoxin involved in defense mechanisms and glutathione metabolism, was detected through candidate gene analysis [50]. Glutathione, as a substrate for glutathione peroxidase, affected the antioxidant activity [51]. Combining other findings and the results of this study, we speculated that Na_2_SeO_3_ treatment increased 1-Cys peroxiredoxin expression and affected the GPx activity by regulating glutathione metabolism, improving the antioxidant system, and thus promoting plant growth and development.

### 3.3. Physiological Mechanism by Which Se Increases the Yield of Tartary Buckwheat

Research has shown that Se can improve the chlorophyll content, photosynthesis, growth and development, yield, quality, and stress resistance in plants [14,22,23,52,53]. In this study, Na_2_SeO_3_ significantly increased the photosynthetic pigment content (Figure 1). Lanza and Dos Reis (2021) found that Se application at low concentrations enhanced the chlorophyll content and photosynthesis in plants [54]. In plants, chloroplasts are the main site of photosynthesis, as well as that of Se reduction and assimilation [55]. Chlorophyll converts light energy into chemical energy during photosynthesis. An increase in the chlorophyll content signifies an increase in the light energy converted by the plant [56]. Golob et al. (2016) reported that Se can increase the photochemical efficiency of photosystem II and electron transport system activity in Tartary buckwheat [19]. Therefore, an increase in chlorophyll content may promote photosynthesis, and the promotion of photosynthesis is directly reflected in the increase in the accumulation of photosynthetic products. Sucrose is the main product of photosynthesis. At 20 and 30 days after Na_2_SeO_3_ application, the sucrose content of the leaves and grains increased significantly (Figure 2). Sucrose in leaves can be transported to grains and then form starch. On the 20th and 30th days, the starch content of grain under the Se6.0 treatment was significantly higher than that of CK (Figure 2). In mature grains, the total starch content was also significantly higher than that of CK (Figure 3). As starch is the main product in the grain-filling process, these results indicate that Se application effectively promotes grain filling, thereby increasing the 1000-grain weight and the number of grains per plant, ultimately promoting Tartary buckwheat yield (Figure 3). Combined with the correlation analysis among the above traits (Figure S3), this study found that Na_2_SeO_3_ application promoted photosynthesis by increasing the formation of photosynthetic pigments, thereby promoting grain filling, increasing the 1000-grain weight and the number of grains per plant, and ultimately promoting the yield of Tartary buckwheat. This conclusion is corroborated by the findings of previous studies [12,20,21,52,57].

Based on the DEGs and core gene analysis, we detected 11 candidate genes regulating chlorophyll accumulation, photosynthesis, sucrose and starch transport, and metabolism. Four of them were novel. FtPinG0006789900.01 is the chlorophyll synthase (CHLG), which is involved in the chlorophyll biosynthetic process and coenzyme transport and metabolism [58]. CHLG was shown via transgenic analysis to participate in the last step of the chlorophyll biosynthetic pathway in tobacco [59]. In the present study, the leaves had a higher chlorophyll content and a high *CHLG* expression level under Na_2_SeO_3_ treatment (Figure 1, Table 1). In addition, CHLG was classified in the black module, which was associated with photosynthetic pigment content based on the WGCNA. Therefore, we suspect that CHLG regulates chlorophyll biosynthesis in Tartary buckwheat. FtPinG0001818900.01 seems to be homologous to probable zinc metallopeptidase EGY3, which is involved in chloroplast development [60,61]. Previous reports showed that both EGY1 and EGY2 are involved in chloroplast development [62,63]. EGY3 is located in chloroplasts and plays an important role in the stabilization of other chloroplast proteins [60]. In the present study, *EGY3* showed a high expression level under Na_2_SeO_3_ treatment and was contained in the black module, which was associated with the photosynthetic pigment content. Although there is no direct evidence to demonstrate that EGY3 is related to chloroplast development, previous studies of the EGY family combined with our results indicate that EGY3 affects chloroplast development. FtPinG0002326800.01 is chlorophyll a-b binding protein 3C (CAB3C) and it regulates photosynthesis and light harvesting in photosystem I [64,65,66]. CAB3C was classified in the light cyan module as associated with starch content in leaves during the grain filling stage. Starch is one of the main stored forms of carbohydrates produced by plants through photosynthesis. Starch accumulation is directly affected by the efficiency of photosynthesis. Therefore, CAB3C may regulate photosynthesis and influence starch synthesis. NewGene_1970 is glyoxylate/hydroxypyruvate reductase 3 (HPR3), and it is associated with the oxidative photosynthetic carbon pathway and photorespiration [67]. Photorespiration and photosynthesis are important biochemical processes in plants, and they influence each other through ribulose bisphosphate carboxylase/oxygenase [68]. In the present study, HPR3 was downregulated under the Na_2_SeO_3_ treatment (Table 1). We speculated that HPR3 regulated photorespiration, thus affecting photosynthesis. The correlation between photorespiration and photosynthesis has been well demonstrated [69,70,71]. NewGene_6299 is the protein CURVATURE THYLAKOID 1B (CURT1B), and it is related to photosynthetic electron transport in photosystem I [72]. CURT1B optimizes photosynthesis under changing light conditions by regulating the structure and dynamics of thylakoid membranes in *Arabidopsis* [73]. CURT1 plays an important role in fine-tuning photosynthesis and plant fitness through changes in thylakoid membrane curvature induced by light [74]. In this study, *CURT1B* showed a high expression level in Tartary buckwheat leaves, indicating that it regulates photosynthesis in the same way as in other species. FtPinG0003227000.01 is starch synthase 3 (SS3), which regulates the starch biosynthetic process. Previous studies have shown that SSIII was involved in starch assembly and transient starch biosynthesis in *Arabidopsis* [75]. SSIIIa regulates starch biosynthesis in the rice endosperm [76]. A high *SS3* expression level was included in the light cyan module, which was associated with the starch content, indicating that SS3 regulates starch synthesis in Tartary buckwheat. FtPinG0004638100.01 is sucrose synthase 5 (SUS5), which controls the sucrose metabolic process. SUS5 has a specific function in callose synthesis in *Arabidopsis* [77]. Yao et al. (2020) postulated that SuSy5 could be involved in providing energy for sucrose retrieval [78]. In rice, SUS is a key enzyme regulating the conversion of sucrose into starch during grain filling [79]. These results suggest that SUS5 may participate in multiple regulatory pathways, including sucrose metabolism. In addition, the present study showed that SUS5 was associated with the sucrose content based on the WGCNA. FtPinG0006796200.01 is inactive beta-amylase 9 (BAM9), which is associated with the polysaccharide catabolic process and starch and sucrose metabolism based on function annotation. BAM9 has been shown to regulate starch degradation in *Arabidopsis* and banana [80,81]. Based on the WGCNA, BAM9 was downregulated in leaves and associated with the starch content, suggesting that BAM9 is involved in starch metabolism in Tartary buckwheat. FtPinG0003149600.01 and NewGene_11121 are SWEET12 (sugars that will eventually be exported transporter 12) and SWEET1, respectively, both belonging to the SWEET sugar transporter family. There is evidence showing that the *SWEET1* and *SWEET12* genes are involved in sucrose transport [82,83,84]. SWEET1 and SWEET12 are included in the black module associated with the sucrose and starch contents during the filling stage. We thus hypothesized that they regulate sucrose transport from leaves to grains in Tartary buckwheat. NewGene_6149 is Glucose-6-phosphate/phosphate translocator 1 (GPT1), which transports Glc6P into plastids of heterotrophic tissues for starch biosynthesis in *Arabidopsis* [85]. Kammerer et al. (1998) reported that glucose 6-phosphate imported via GPT could be used for starch biosynthesis [86]. In sweet potatoes, the *IbG6PPT1* gene might play a critical role in the distribution of carbon sources in the source and sink and the accumulation of carbohydrates in storage tissues [87]. Based on these findings, it was speculated that GPT1 affects starch biosynthesis by transporting sucrose in Tartary buckwheat.

### 3.4. A Hypothetical Regulation Model of the Genes Responding to Se in Tartary Buckwheat

**Figure 7:**
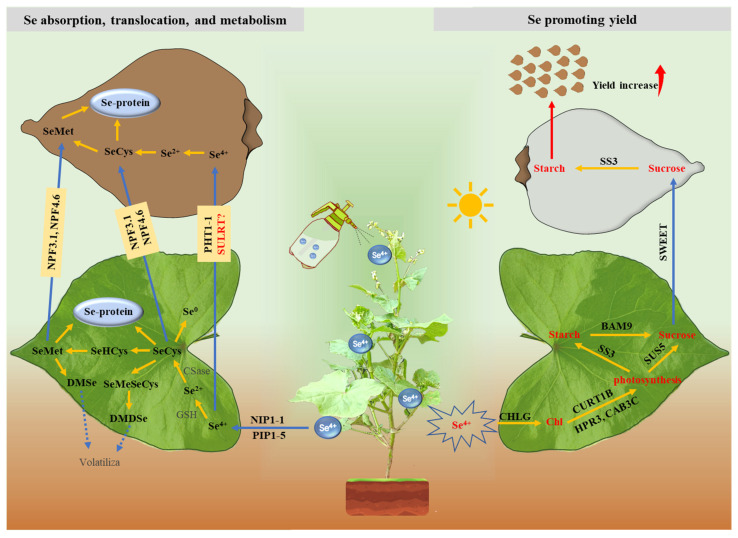
A hypothetical regulation model of the genes responding to Na_2_SeO_3_ in Tartary buckwheat. The left side of the figure illustrates the key regulatory genes and pathways involved in selenite absorption, translocation, and metabolism in leaves and grains. The right side of the figure shows the key regulatory genes and pathways that promote photosynthesis, grain filling, and increased yield under selenite treatments. The yellow arrows indicate the transformation between substances, the blue arrows indicate substance transport, and the red arrow indicates a result.

A hypothetical Se absorption, translocation, metabolism, and yield improvement model was constructed based on WGCNA, core genes, transcript expression, annotation, and trait correlation analyses (Figure 7). Selenite (Na_2_SeO_3_) was sprayed on the leaves of Tartary buckwheat at the initial flowering stage. It is regulated by the aquaporins NIP1-1 and PIP1-5 and enters mesophyll cells through passive absorption. Then, it participates in glutathione metabolism through 1-Cys peroxiredoxin regulation, takes part in Se compound metabolism through MGL regulation, and finally forms seleno-amino acids, selenoproteins, and elemental Se. Some seleno-amino acids are also regulated by NPF3.1 and NPF4.6 of the NRT1/PTR family, transported from mesophyll cells to grains, and then metabolized. Some unmetabolized selenite can be transported into grains through phosphate transporter PHT1-1 or sulfate transporters SULTR3;1 and SULTR3;4. They are then metabolized in the grains to form seleno-amino acids and selenoproteins.

Se assimilation and metabolism are carried out in chloroplasts, and chlorophyll is located on the thallus membrane of chloroplasts. Chloroplasts are the main sites of photosynthesis. Therefore, external Se application has a certain effect on chlorophyll and photosynthesis in Tartary buckwheat, and this effect has also been widely confirmed in other species [11,14,20,21,22,88]. We speculated that exogenous selenite enhances the expression of the *EGY3* and *CHLG* genes, which regulate chloroplast development and promote chlorophyll synthesis. Chlorophyll synthesis may improve plants’ ability to convert light energy and promote photosynthesis. Photosynthesis is positively regulated by CURT1B, HPR2, and CAB3C and produces carbohydrates. High *SS3* expression regulates transitory starch formation in leaves. Transitory starch is then degraded into sucrose, regulated by BAM9. The synthesis of sucrose, the main photosynthetic product, is controlled by SUS5. Sucrose enters the grains via the transporters SWEET1, SWEET12, and GPT1, where it forms storage starch with SS3 regulation. This process promotes grain filling, increases the 1000-grain weight and grain number, and improves the yield of Tartary buckwheat.

## 4. Materials and Methods

### 4.1. Plant Materials

Tartary buckwheat cultivar ‘Xiqiao 1’ (XQ 1) was used in this study. It is an early-maturing, high-yielding commercial variety widely grown in southwest China. Seeds were provided by the Key Laboratory of Coarse Cereal Processing, Ministry of Agriculture and Rural Affairs, Chengdu University (Chengdu, Sichuan province, China). Sodium selenite (Na_2_SeO_3_) was used in this study and it was purchased from Alfa Essa (China) Chemical Co., Ltd. (Shanghai, China).

### 4.2. Experimental Design

The experiment was conducted at the experimental field station in Xinshi Town, Jianyang City, Sichuan Province (30°19′11.9″ N, 104°33′15″ E). The plot dimensions were 2 m × 3 m (width × length), and the interval was 1 m between plots. In each hole, 10–12 plants were sown. The distance between the holes was 0.25 m. Plants were watered and managed following standard local practices. Four concentrations of Na_2_SeO_3_ were used, namely 0 (CK), 1.5 (Se1.5), 3.0 (Se3.0), and 6.0 mg/L (Se6.0). Plots were set up in a randomized complete block design with three replications. Na_2_SeO_3_ treatments were carried out by spraying on the foliage at the initial flowering stage (46 days after sowing). The spraying time was 4–6 p.m. on a windless and cloudy day, and the plots were sprayed with a hand-shaking sprayer until the liquid started to drip from the leaves.

### 4.3. Measurement of Photosynthetic Pigment Content

The contents of photosynthetic pigments, including Chl *a*, Chl *b*, and Car, were determined by spectrophotometry [89]. The 7th leaves were collected and measured as the main functional leaves [90]. From each treatment, 0.2 g of fresh leaves was selected at 10, 20, and 30 days after Na_2_SeO_3_ treatment, cut into small pieces, soaked in 95% ethanol solution, and placed in the dark for 48 h. Filtrates were determined using a Synergy™ HTX Multi-Mode Microplate Reader (BioTek Instruments Inc., Winooski, VT, USA) at wavelengths of 470, 665, and 645 nm. The total Chl content was calculated as the sum of Chl *a* and Chl *b* contents.

### 4.4. Identification of Sucrose and Starch Content, and Glutathione Peroxidase Activity

At 10, 20, and 30 days after Na_2_SeO_3_ treatment, grain and leaf samples were collected to measure the sucrose and starch contents and GPx activity. They were assessed using a sucrose measurement kit, starch content test kit, and glutathione peroxidase (GSH-PX) assay kit (Colorimetric method) (Jiancheng Bioengineering Institute, Nanjing, China) following the manufacturer’s protocols.

### 4.5. Investigation of Agronomic and Yield-Related Traits

At maturity stage, the data on plant height, stem diameter, branch number on the main stem, and main stem node number for agronomic traits and the grain number per plant, grain weight per plant, and 1000-grain weight were collected from five plants in each plot. Plot yield refers to the total weight of grains from all the plants in it. The plot yield was finally converted into yield per hectare.

### 4.6. Determination of Grain Quality and Se Content

After harvest, grains were dried and ground to powder with a diameter of 0.25 mm for substance extraction. The total starch, amylose, and flavonoid contents were determined using a starch content test kit, amylose colorimetric assay kit, and plant flavonoids test kit (Jiancheng Bioengineering Institute, Nanjing, China) following the manufacturer’s protocols. The amylopectin content was the value of the total starch minus the amylose content.

The total Se and inorganic Se contents of grains were measured after harvest. The grains were dried and ground to powder with a diameter of less than 0.15 mm. To determine the Se content, 0.2 g of each sample was digested using a microwave digestion apparatus (APL Touchwin 2.0, Hunan, China) with 5 mL of nitric acid solution, suspended in 50 mL of ultrapure water, filtered, and analyzed using inductively coupled plasma-mass spectroscopy (ICP-MS) (iCAP RQ, Thermo Fisher Scientific, Waltham, MA, USA). Hydride Generation Atomic Fluorescence Spectrometry (HG-AFS) was used to measure the inorganic Se content. For hydrochloric acid hydrolysis, 0.25 g of each sample was mixed with potassium ferrocyanide solution and n-octanol. The values were tested using an Atomic Fluorescence Spectrophotometer (AFS-8520, Haiguang Instruments, Beijing, China). The organic Se content was determined as the total Se minus the inorganic Se content. The conversion rate of organic Se was calculated as the percentage of organic Se of the total Se.

### 4.7. RNA Isolation and Sequencing

At 10, 20, and 30 days after Na_2_SeO_3_ treatment, the grain and leaf tissues were sampled with 3 independent biological replicates. The total RNA of each sample was extracted using a Plant RNA Extraction Kit (Takara Bio, Otsu, Japan) following the manufacturer’s protocol. RNA concentration and purity were measured using NanoDrop 2000 (Thermo Fisher Scientific, DE, USA). RNA integrity was assessed using an RNA Nano 6000 Assay Kit for the Agilent Bioanalyzer 2100 system (Agilent Technologies, Santa Clara, CA, USA). The RNA with an RIN larger than 8.5 was used to construct complementary DNA (cDNA) libraries. Paired-end 2 × 150 bp reads were generated using the Illumina platform (NovaSeq 6000, Illumina Inc., San Diego, CA, USA).

### 4.8. Transcriptome Assembly, DEGs, and Annotation Analysis

The adapters of the obtained sequence reads, ploy-N (>10%), and low-quality reads (the base number of Q ≤ 10 greater than 50%) were removed from the raw sequences to generate clean sequences of high quality. The clean reads were mapped to the reference genome sequences of Tartary buckwheat (http://www.mbkbase.org/Pinku1/ (accessed on 14 August 2018)) using Hisat2 v 2.0.4 software [91]. The mapped reads were assembled using StringTie v1.3.4d software [92], and the gene expression values (FPKM) were calculated [93]. The DEGs were identified using the R package DESeq2 v 1.6.3 with a model based on the negative binomial distribution [94]. The screening criteria for DEGs were as follows: FDR < 0.01 and FC ≥ 2. Compared with the reference genome annotation, the unannotated transcript regions are defined as novel transcripts or new genes. The gene functions were annotated based on databases including Nr (NCBI non-redundant protein sequences), Pfam (Protein family), KOG/COG (Clusters of Orthologous Groups of proteins), Swiss-Prot (a manually annotated and reviewed protein sequence database), KO (KEGG Ortholog database), and GO (Gene Ontology). KOBAS was used to analyze the statistical enrichment of differentially expressed genes in KEGG pathways [95].

### 4.9. WGCNA of Physiological Index in Leaves and Grains

The WGCNA package in R was used to analyze gene co-expression modules and the correlation between the eigengene modules and traits [96]. Genes with FPKM ≥ 0.001 and variation in FPKM ≥ 0.5 were used. The minimum module size was 30, and the minimum height for merging modules was 0.25. The optimal soft threshold (β) was determined using the pickSoftThreshold function in the WGCNA package to make the network converge infinitely to the distribution of the scale-free network.

### 4.10. qRT-PCR Validation

qRT-PCR was used to verify the expression level of candidate genes in response to selenite in the leaves and grains of Tartary buckwheat. Primers were designed using an online system (https://www.ncbi.nlm.nih.gov/tools/primer-blast/ (accessed on 27 July 2010)). The cDNA was synthesized using the SweScript One-Step gDNA Remover and cDNA Synthesis SuperMix Kit (Servicebio, Wuhan, China). The qRT-PCR reaction was performed in a qTOWER^3^G real-time PCR system (Analytik Jena AG, Jena, Germany) using the following procedure: 95 °C for 30 s, followed by 40 cycles of 95 °C for 10 s, 58 °C for 30 s, and 72 °C for 30 s. The housekeeping gene *FtActin* was used as a reference [97]. Gene expression was normalized using the 2^−ΔΔCt^ method [98].

### 4.11. Statistical Analysis

Microsoft Excel 2016 (Microsoft, Redmond, WA, USA) was used to process and analyze the data. The SPSS v 20.0 (IBM, Inc., Armonk, NY, USA) data processing system was used to analyze the Pearson correlation coefficients between traits and the significant differences based on one-way ANOVA. Origin 2022 (Origin Lab Corp., Northampton, MA, USA) was utilized to draw figures.

## 5. Conclusions

This study found that a Na_2_SeO_3_ concentration around 6.0 mg/L was the best for Se enrichment and a yield increase in Tartary buckwheat, which can guide the production demonstration of selenium-enriched Tartary buckwheat. Phenotypic traits and transcriptome analysis revealed the regulatory genes by which Se promoted a yield increase and Se uptake, transport, and metabolism in Tartary buckwheat for the first time. Both aquaporins (NIP1-1 and PIP1-5) and an inorganic phosphate transporter (PHT1-1) regulated selenite absorption and transport, respectively. The proton-dependent oligopeptide transporters NPF3.1 and NPF4.6 control organic Se transport. MGL is involved in the selenocompound metabolism. Additionally, selenite activated the upregulation of *CHLG* and *CURT1B*, which increased chlorophyll synthesis and the photoelectron transport ability, improved photosynthesis, increased sucrose synthesis and transport in leaves and starch synthesis and accumulation in grains, promoted grain filling, and thus increased Tartary buckwheat yield. This was regulated by photosynthesis and sucrose and starch metabolism-related genes, including *CAB3C*, *HPR3*, *SUS5*, *BAM9*, *SS3*, *SWEET1*, and *SWEET12*. These results lay a theoretical foundation for the functional studies of genes related to selenite absorption, transport, and metabolism, as well as genes associated with photosynthesis and yield, and their utilization in Tartary buckwheat breeding.

## Figures and Tables

**Table 1:** Candidate genes regulating Se enrichment and the yield increase.

Gene ID	Log_2_ (Fold Change)	Gene/Protein Name	Annotation
FtPinG0000179900.01	1.114392	Protein NRT1/PTR FAMILY 4.6, NPF4.6	Nitrate assimilation; amino acid transport and metabolism.
FtPinG0001747900.01	−1.37391	Methionine gamma-lyase, MGL	Selenocompound metabolism; cysteine and methionine metabolism.
FtPinG0001818900.01	1.571489	Probable zinc metallopeptidase EGY3	Chloroplast development; response to salt stress.
FtPinG0002326800.01	1.03773	Chlorophyll a-b binding protein 3C, CAB3C	Photosynthesis; light harvesting in photosystem I; response to light stimulus.
FtPinG0003149600.01	−1.16556	Bidirectional sugar transporter SWEET12	Sucrose transport.
FtPinG0003227000.01	1.185321	Starch synthase 3, SS3	Starch and sucrose metabolism.
FtPinG0003351100.01	3.213713	Aquaporin NIP1-1	Facilitates the transport of water and small neutral solutes across cell membranes.
FtPinG0004638100.01	2.746147	Sucrose synthase 5, SUS5	Starch and sucrose metabolism.
FtPinG0006789900.01	3.461694	Chlorophyll synthase CHLG	Chlorophyll biosynthetic process; coenzyme transport and metabolism.
FtPinG0006796200.01	−1.05487	Inactive beta-amylase 9, BAM9	Polysaccharide catabolic process; starch and sucrose metabolism.
FtPinG0007417400.01	1.154844	Inorganic phosphate transporter 1-1, PHT1-1	Inorganic ion transport and metabolism; carbohydrate transport and metabolism; arsenate ion transmembrane transport.
FtPinG0007446600.01	1.289527	1-Cys peroxiredoxin	Glutathione metabolism; defense mechanisms.
FtPinG0007572400.01	−1.44642	Sulfate transporter 3.1, SULTR3;1	Sulfate transport; inorganic ion transport and metabolism.
NewGene_11121	−2.32612	Bidirectional sugar transporter SWEET1	Sugar efflux transporter for intercellular exchange.
NewGene_1970	−2.47558	Glyoxylate/hydroxypyruvate reductase HPR3	Oxidative photosynthetic carbon pathway; photorespiration.
NewGene_2911	1.604918	Probable aquaporin PIP1-5	Facilitates the transport of water and small neutral solutes across cell membranes.
NewGene_3379	1.192383	Protein NRT1/PTR FAMILY 3.1, NPF3.1	Nitrate assimilation; regulation of nitrite uptake into higher plant chloroplasts; amino acid transport and metabolism.
NewGene_6149	1.764971	Glucose-6-phosphate/phosphate translocator 1, GPT1	Transports Glc6P into plastids of heterotrophic tissues where it can be used as a carbon source for starch biosynthesis.
NewGene_6299	2.034421	Protein CURVATURE THYLAKOID 1B, CURT1B	Photosynthetic electron transport in photosystem I.
NewGene_9793	−1.23295	Probable sulfate transporter 3.4, SULTR3;4	Phosphate ion transport; sulfate transport; inorganic ion transport and metabolism.

## Data Availability

The RNA-seq data are deposited in the BioProject database of NCBI under the accession number PRJNA1201341.

## Supplementary Materials

The following supporting information can be downloaded at https://www.mdpi.com/article/10.3390/plants14030423/s1: Figure S1: Heat map of gene expression level correlation; Figure S2: Soft threshold determination of gene co-expression network; Figure S3: Correlation coefficient analysis among physiological traits during the filling stage, agronomic, yield-related, and quality traits and Se enrichment. Table S1: Quality of RNA-seq reads of Tartary buckwheat samples. Table S2: Primers of candidate genes for quantitative real-time polymerase chain reaction used in this study.
