# Supplementary material for: Weighted Gene Correlation Network Analysis Reveals Key Regulatory Genes Influencing Selenium Enrichment and Yield with Exogenous Selenite in Tartary Buckwheat
Source: Plants (Basel). 2025 Feb 1;14(3):423. doi: 10.3390/plants14030423 (PMC11820427; doi:10.3390/plants14030423)
Supplement: Supplementary file 1 [file plants-14-00423-s001.zip › plants-3413324-supplementary.pdf]

Supplementary Figures

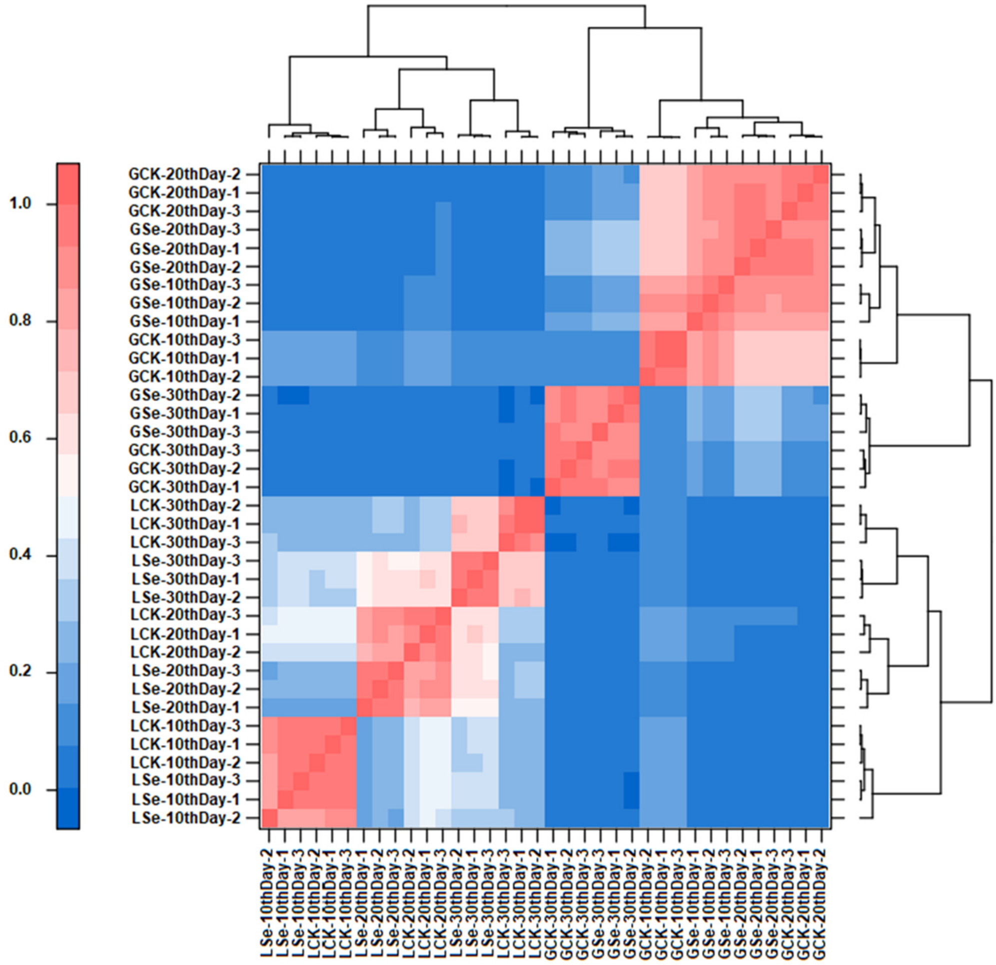

Figure S1. Heat map of gene expression level correlation. GCK, control of grains; GSe, grains with Na_2SeO_3 treatment of 6.0 mg/L; LCK, control of leaves; LSe, leaves with Na_2SeO_3 treatment of 6.0 mg/L; The 10thDay, 20thDay, and 30thDay indicate the days after selenite treatments.

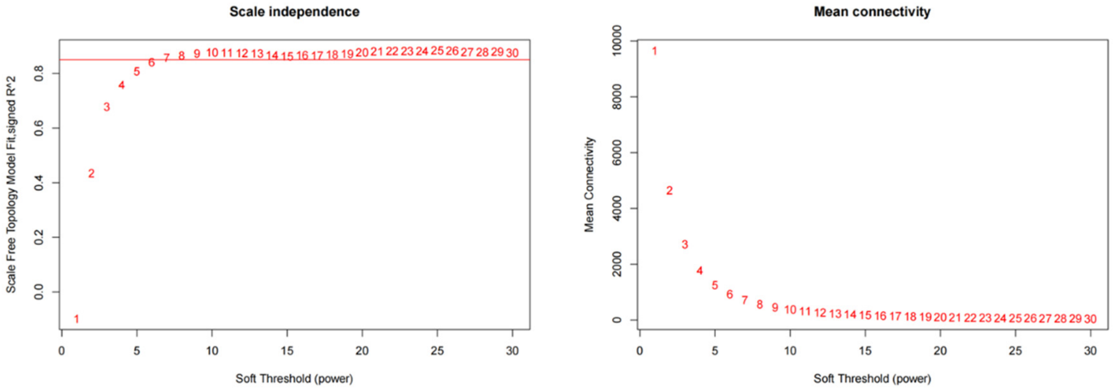

Figure S2. Soft threshold determination of gene co-expression network.

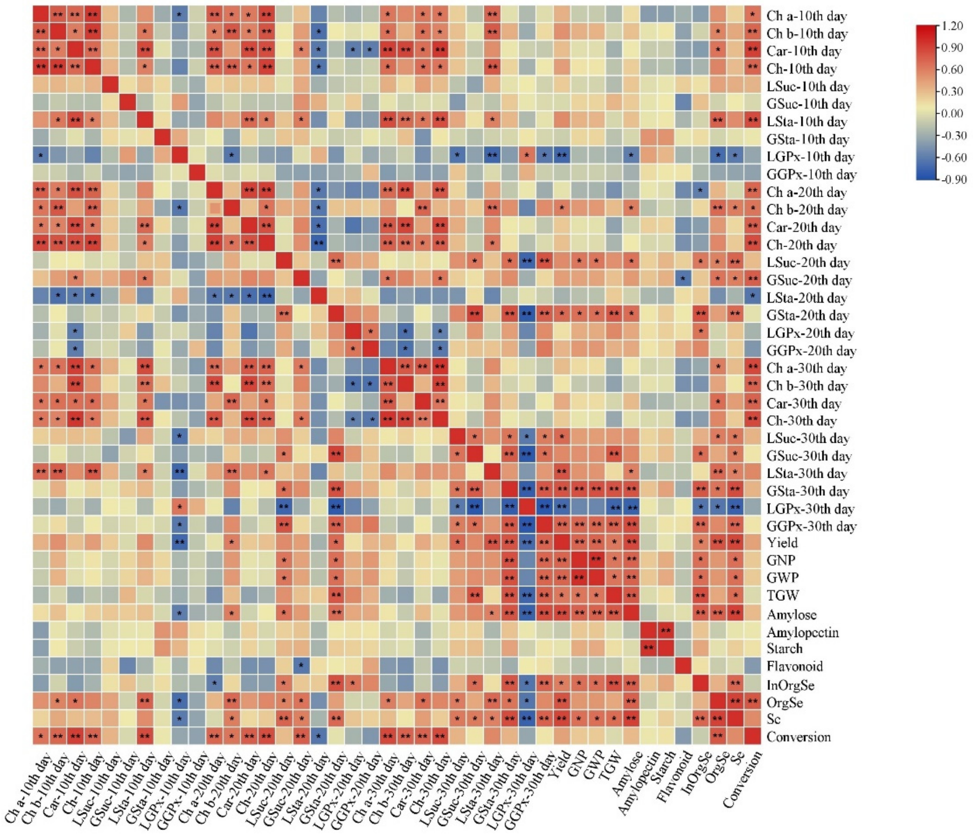

Figure S3. Correlation coefficient analysis among physiological traits during the filling stage, agronomic, yield-related, and quality traits and Se enrichment. Ch, chlorophyll; Car, carotenoid; LSuc, leaf sucrose content; GSuc, grain sucrose content; LSta, leaf starch content; GSta, grain starch content; LGPx, glutathione peroxidase activity of leaves; GGPx, glutathione peroxidase activity of grains; GNP, grain number per plant; GWP, grain weight per plant; TGW, 1000-grain weight; InOrgSe, inorganic selenium content; OrganSe, organic selenium content.

Supplementary Tables

Table S1 Quality of RNA-seq reads of Tartary buckwheat samples.

Samples	Clean reads	Clean bases	GC Content	%≥Q30
GCK-10thDay-1	24,370,613	7,296,201,048	45.22%	95.87%
GCK-10thDay-2	23,742,903	7,110,206,856	45.15%	95.64%
GCK-10thDay-3	24,302,811	7,276,991,778	45.19%	96.07%
GSe-10thDay-1	19,512,524	5,844,440,644	45.60%	95.60%
GSe-10thDay-2	23,174,726	6,937,099,688	45.31%	95.95%
GSe-10thDay-3	20,435,004	6,118,112,990	45.13%	96.20%
GCK-20thDay-1	22,493,713	6,736,439,202	45.42%	95.88%
GCK-20thDay-2	24,718,741	7,402,221,442	45.54%	96.00%
GCK-20thDay-3	23,715,395	7,099,289,762	45.50%	96.24%
GSe-20thDay-1	23,230,705	6,956,799,418	45.41%	95.96%
GSe-20thDay-2	22,554,199	6,751,534,196	45.49%	95.89%
GSe-20thDay-3	22,379,402	6,701,487,684	45.54%	95.52%
GCK-30thDay-1	19,973,766	5,979,250,966	46.81%	95.26%
GCK-30thDay-2	19,185,318	5,743,296,198	46.11%	94.75%
GCK-30thDay-3	21,162,634	6,334,196,818	46.80%	96.05%
GSe-30thDay-1	21,370,282	6,398,332,182	46.43%	95.62%
GSe-30thDay-2	20,183,593	6,042,851,068	46.59%	95.05%
GSe-30thDay-3	20,237,308	6,057,150,446	47.22%	95.40%
LCK-10thDay-1	21,055,555	6,303,495,866	46.20%	95.61%
LCK-10thDay-2	21,208,981	6,351,459,032	46.54%	94.96%
LCK-10thDay-3	24,073,816	7,208,134,038	46.44%	95.04%
LSe-10thDay-1	20,727,770	6,209,190,526	45.83%	94.98%
LSe-10thDay-2	27,152,351	8,111,389,582	46.63%	95.19%
LSe-10thDay-3	21,415,837	6,414,537,118	45.91%	94.65%
LCK-20thDay-1	22,484,057	6,733,291,520	45.23%	96.09%
LCK-20thDay-2	19,841,946	5,943,006,914	46.29%	95.33%
LCK-20thDay-3	21,975,852	6,581,962,724	44.90%	93.17%
LSe-20thDay-1	23,663,075	7,087,915,458	44.41%	94.04%
LSe-20thDay-2	21,305,397	6,378,360,826	44.97%	95.60%
LSe-20thDay-3	21,016,792	6,294,196,230	44.78%	95.71%
LCK-30thDay-1	22,587,537	6,764,547,648	44.70%	95.86%
LCK-30thDay-2	22,933,226	6,868,036,186	44.69%	95.53%
LCK-30thDay-3	20,484,358	6,133,103,578	45.08%	95.77%
LSe-30thDay-1	21,330,303	6,383,690,318	44.80%	95.44%
LSe-30thDay-2	19,719,553	5,906,227,894	44.99%	95.09%
LSe-30thDay-3	20,828,312	6,238,811,406	44.78%	94.89%

GCK, control of grains; GSe, grains with Na_2SeO_3 treatment of 6.0 mg/L; LCK, control of leaves; LSe, leaves with Na_2SeO_3 treatment of 6.0 mg/L; The 10thDay, 20thDay, and 30thDay indicate the days after selenite treatments.

Table S2 Primers of candidate genes for quantitative real-time polymerase chain reaction used in this study

Gene ID	Primers	
	**Forward sequence**	**Reverse sequence**
FtPinG0000179900.01	TACCGATATTCGCCAGCACC	TTCATGGTTGCGGCTTGTTG
FtPinG0001747900.01	GGCCATCAAGTTTGGTGAACTC	GGCTACTTTGGCGTTCATTGTT
FtPinG0001818900.01	CACCATTGGAAGACGACAGGTA	TGAAGTAAGGCGAACCGAAGAA
FtPinG0002326800.01	GGAGTCAAGTTCGGAGAGGC	GATCAAGCTCGGGTTTCCCA
FtPinG0003149600.01	CCTCGTCTATCTCGCACCAC	AAATCGCGCACACATAAGGC
FtPinG0003227000.01	ATAGGAGCTCTGGTCCTCTTGT	CACCAGTCAGCATCCTTCTTCT
FtPinG0003351100.01	CTATTGGGATCGACGTTGGCTA	TCGACGACAAAGGATTGGAGTT
FtPinG0004638100.01	AGCATGGTCCAAGGATGAAAGT	AAGAACTTCGACACGTAACCGA
FtPinG0006789900.01	AAGCTGCAAAATGGATTTGCGT	AACCTGAGGCAATACCAAACCT
FtPinG0006796200.01	GAAGATGGTGTGGTGGATCAGT	GTGAACACCCGGAAGTTCTTTG
FtPinG0007417400.01	AACACTGGCAAGAGAAGGAACA	GGGGCCAAAGTTAGCAAAGAAG
FtPinG0007446600.01	AAGCTTCATGATTTCATCGGCG	GCTTGACTCCTCTCTTGGTGAA
FtPinG0007572400.01	AAACCTTCTTCCCCGACGAC	CCCACTCAAAGAACGGGACA
NewGene_11121	CCCACGGTGACATTTAAGAGGA	ATACCAAGCGGATAAGAGGCAG
NewGene_1970	TCATAAACATAGGCCGAGGAGC	TTCGTTCTCAAACACGTCAAGC
NewGene_2911	GCTTGGCATGACCATTGGATTT	GATCACCACTTGGTGGTACAGT
NewGene_3379	AGAAGGGTGGCATGATCACAAT	CTTTGGTTAACGGCATGTGGAG
NewGene_6149	CCCCTACCCTTGGTTAACTTCC	CAGTACTGGGTGTCTCAGCAAT
NewGene_6299	CCGGCAGAAGTTGCTGTAGT	CCACACCCACTGTGCTTACT
NewGene_9793	ACGGCTCATACTTGGCAGTC	GAAGAGCAAGATCCGGCCAT
